# The complete mitochondrial genome of *Anatrichus pygmaeus* Lamb, 1918 (Diptera, Chloropidae)

**DOI:** 10.1080/23802359.2022.2097029

**Published:** 2022-07-12

**Authors:** Xiaodong Cai, Ding Yang, Xiaoyan Liu

**Affiliations:** a Department of Entomology, College of Plant Protection, China Agricultural University, Beijing, China; b Hubei Insect Resources Utilization and Sustainable Pest Management Key Laboratory, College of Plant Science and Technology, Huazhong Agricultural University, Wuhan, China

**Keywords:** Mitochondrial genome, Carnoidea, Chloropidae, *Anatrichus pygmaeus*, phylogenetics

## Abstract

The genus *Anatrichus* Loew, 1860 belongs to the subfamily Oscinellinae of the family Chloropidae. We report the complete mitogenome of *Anatrichus pygmaeus* as the new representative of the subfamily Oscinellinae. The complete mitochondrial genome was 17,125 bp in length. It consisted of 13 protein-coding genes, 22 transfer RNA genes, two ribosomal RNA genes, and a control region. The phylogenetic result showed that the family Chloropidae is monophyletic, and the subfamily Chloropinae is a sister group of the subfamily Oscinellinae.

The genus *Anatrichus* Loew, 1860 belongs to the subfamily Oscinellinae (Kanmiya 1983; Nartshuk 2010) and is a small genus of Chloropidae. It can be easily separated from other genera of Chloropidae by the following features: scutellum covered in long straight spinescent setae; abdominal tergites 1 + 2 + 3 fused into a large plate covering the abdomen, pear-shaped (Kubík and Barták 2010; Mlynarek and Wheeler 2018). *Anatrichus* is an Old World genus occurring in the Afrotropical, Oriental, Australian, and Palearctic Realms (Paramonov 1961; Mlynarek and Wheeler 2018). There are only five valid species [*Anatrichus bimaculatus* (Kubík and Barták 2010); *Anatrichus erinaceus* Loew, 1860; *Anatrichus hystrix* (Kertesz, 1914); *Anatrichus pygmaeus* Lamb, 1918; *Anatrichus taprobane* (Andersson, 1977)] in the world, and only one species (*A. pygmaeus*) was recorded in China until now (Kubík and Barták 2010; Mlynarek and Wheeler 2018). The feeding habits of *Anatrichus* are not clear (Sabrosky 1961). Some biological records of its larvae are related to several stem-boring Lepidoptera on rice. For example, some larvae of *A. pygmaeus* were found feeding on dead stem borer larvae in a rice plant and regarded as parasites (Wongsiri et al. 1974). Some authors reported that the larvae of *A. pygmaeus* fed on the decomposing plant tissue and believed that the larvae were saprophagous (Sabrosky 1961; Nartshuk 2014).

The specimens of *A. pygmaeus* were collected from Mengla (101°33′E, 21°28′N), Xishuangbanna, Yunnan by Liang Wang on 24 November 2020, and then identified by Xiaodong Cai. The specimens were preserved in 95% ethanol and stored at −20 °C refrigerator in the Entomological Museum of China Agricultural University (Voucher number: CAUcxd20220010030, Liang Wang, 1352659341@qq.com). The total DNA was extracted from the adults’ muscle tissue using DNeasy Blood & Tissue Kit (Qiagen, Germany). All quantified DNA extracts were included in a single pool at equimolar concentration, aiming for 50 ng/µL of dsDNA per sample, resulting in a DNA pool of ∼5 µg. The library was sequenced on an Illumina NovaSeq 6000 by Novogene. Raw read data was filtered and trimmed in Trimmomatic v0.30 (Bolger et al. 2014). About 6GB of high-quality data was used to assemble the mitochondrial genome with the *de novo* assembler IDBA-UD (Peng et al. 2012). The bait sequence (COI) was amplified by standard PCR reactions. And a BLAST search was carried out with BioEdit 7.0.5.3. The complete mitochondrial genome sequence was annotated by MITOS (Bernt et al. 2013) and checked manually by Geneious v.9.0.2 (Kearse et al. 2012). The tRNA genes were identified by MITOS and rechecked using the tRNAscan-SE web server (Schattner et al. 2005).

The complete mitochondrial genome of *A. pygmaeus* (GenBank accession: OM214541) was 17,125 bp in length. It contained 37 typical insect mitochondrial genes (13 protein-coding genes, 22 transfer RNA genes, and two ribosomal RNA genes) and a control region. The structure of this mitochondrial genome was similar to other Dipteran flies reported previously (Chen et al. 2020; Pi et al. 2021; Wang et al. 2021; Zhang et al. 2021). Its nucleotide composition was A (41.7%), G (8.0%), C (12.0%), and T (38.3%). Among the 13 protein-coding genes, eleven genes used the ATN as the start codon (6 with ATG, 4 with ATT, 1 with ATC), but *NAD5* started with GTG and *COX1* started with TCG. TAA was used as the stop codon for nine protein-coding genes. However, the *COX1*, *COX2*, *NAD4*, and *NAD5* used a single T as the incomplete stop codon.

The phylogeny analysis based on 18 Diptera species protein-coding genes was performed by the IQ-TREE web server (Trifinopoulos et al. 2016) with 1000 bootstrap replications (Figure 1). Three species of Chloropidae were included in the analysis, of which *Anatrichus pygmaeus* and *Dicraeus orientalis* Becker, 1911 belong to the subfamily Oscinellinae and *Chlorops oryzae* Matsumura, 1915 belongs to the subfamily Chloropinae. The phylogenetic result showed that the monophyly of Chloropidae was strongly supported (100%), which was consistent with previous phylogenetic results based on morphological characteristics (Griffiths 1972). The monophyly of the subfamily Oscinellinae was also strongly supported (100%), and the subfamily Chloropinae is a sister group of the subfamily Oscinellinae.

## Data Availability

Mitochondrial genome sequence can be accessed via accession number OM214541 in GenBank of NCBI at https://www.ncbi.nlm.nih.gov. The associated BioProject, Bio-Sample, and SRA, numbers are PRJNA795831, SAMN24781128, and SRR17640167, respectively. The phylogeny is based on *A. pygmaeus* and other 17 Diptera species, which was performed by ML analysis of the 13 protein-coding genes. “*” indicated newly sequenced data in this study.
